# Effects of Noise on English Listening Comprehension among Chinese College Students with Different Learning Styles

**DOI:** 10.3389/fpsyg.2017.01764

**Published:** 2017-10-16

**Authors:** Xiaohu Yang, Meng Jiang, Yong Zhao

**Affiliations:** ^1^Speech-Language-Hearing Center, School of Foreign Languages, Shanghai Jiao Tong University, Shanghai, China; ^2^Language & Brain Research Center, Sichuan International Studies University, Chongqing, China; ^3^Department of Translation and Interpreting, School of Foreign Languages, Shanghai Jiao Tong University, Shanghai, China

**Keywords:** noise, learning styles, learning modes, listening comprehension, foreign language

## Abstract

This study was intended to determine whether the effects of noise on English listening comprehension would vary among Chinese college students with different learning styles. A total of 89 participants with different learning styles measured using Kolb’s (1985) Learning Style Inventory finished English listening comprehension tests in quiet and in white noise, Chinese two-talker babble, and English two-talker babble respectively. The results showed that the participants in general had significantly poorer performance in the two babble conditions than in quiet and white noise. However, the participants with assimilative and divergent learning styles performed relatively better in Chinese babble, and exhibited stable performance across the three noisy conditions, while the participants with convergent and accommodative learning styles had more impaired performance in both Chinese babble and English babble than in white noise. Moreover, of Kolb’s four learning modes, reflective observation had a facilitative effect on listening performance in Chinese babble and English babble. These findings suggest that differences in learning style might lead to differential performance in foreign language listening comprehension in noise.

## Introduction

As speech communication in everyday conditions often takes place in the presence of various kinds of background noise, the ability to understand speech in noisy backgrounds is a very important skill. Speech communication is often accompanied by both energetic and informational masking (IM) produced by noise. Energetic masking (EM) arises out of the competition between target and masker at the auditory periphery, i.e., overlapping excitation patterns in the cochlea or auditory nerve (Durlach et al., 2003), and can affect speech perception by rendering unavailable potential cues to the identity of segments and their boundaries as well as interfering with access to prosodic cues (Garcia Lecumberri et al., 2010). IM refers to the potentially distracting effect of the masker that can cause interference with decisions at higher levels of processing, thus resulting in an inability to detect target signals embedded in other sounds at the central auditory system even when the signals are clearly audible (Garcia Lecumberri et al., 2010).

Speech communication in noisy background is especially challenging for second language (L2) listeners. Their listening experience outside the classroom is always problematic: conversations in restaurants are difficult to continue; the airport announcement is often inaudible; and the telephone never seems loud enough. Earlier studies have paid considerable attention to L2 listening in noise and examined a range of factors that can cause individual differences in listening performance as reviewed in the following section. The present study specially focused on the role of learning styles in English listening comprehension in different types of noise among Chinese college students. The study may provide unique insights into the sources of individual differences exhibited in L2 listening comprehension in adverse conditions. In addition, by demonstrating how learners’ listening comprehension in different types of noise may vary with their learning styles, the findings of this study may help teachers and students gain a better understanding of the effects of adverse conditions on their listening experience and adopt in turn effective coping strategies in accordance with their personal cognitive features.

### L2 Speech Perception in Noise

L2 listeners often have more difficulties in perceiving and understanding speech in the presence of noise than native listeners. For example, Garcia Lecumberri and Cooke (2006) compared English and Spanish listeners’ perception of English intervocalic consonants in speech shaped noise that was generated by filtering white noise through the long-term spectrum of speech, multi-talker babble (i.e., a mixture of more than one talkers), competing English and Spanish speech (i.e., a single interfering talker). The results indicated that non-native performance fell short of that of native listeners in quiet, and the differences became larger in noisy conditions. Similarly, Cutler et al. (2008) demonstrated that for English consonant identification in quiet and eight-talker babble conditions, the difference between L2 and L1 listeners in the babble noise condition was much greater than in quiet.

Native and non-native listeners had differential performance in different noise types and signal-to-noise ratios (SNRs). It has been shown that the performance of L2 listeners decreased with SNR and became more impaired in white noise that has equal energy for all frequencies, than pink noise in which the energy level decreases while frequency increases, or aircraft noise (Shimizu et al., 2002); speech-shaped noise affected speech perception more than speech from a competing talker at the same SNR (Festen and Plomp, 1990; Garcia Lecumberri and Cooke, 2006); factory noise hindered speech perception more than speech-shaped noise (Cooke and Scharenborg, 2008). Even for multi-speaker babble, large differences were observed as different numbers of competing speakers affected speech perception (Simpson and Cooke, 2005). It is notable that the effect of different types of noise on L1 and L2 listeners’ speech perception was highly variable. According to the results of Broersma and Scharenborg (2010), noisy conditions affected L1 and L2 listeners’ performance differentially only for some consonants, not for all consonant. Moreover, for the consonants for which differences in perceptual performance between L1 and L2 listeners were observed, the effects of noise types were not always the same, and no single type of noise could affect L1 and L2 listeners differently for all of these consonants.

The similarity between speech materials and background maskers is critical to L2 listeners’ speech perception in noise (e.g., Van Engen and Bradlow, 2007; Brouwer et al., 2012; Gautreau et al., 2013; Calandruccio et al., 2014; Calandruccio and Zhou, 2014). It has been shown that the more similar the target speech was acoustically and/or linguistically to the masker, the more effective the masker was. In particular, listeners had better performance when the speech masker was in a different language from the target speech. Actually, the different effects observed between the linguistic maskers could be the results of the spectral differences between the masker conditions (Calandruccio et al., 2010). Furthermore, the difference in performance between L1 and L2 listeners was larger when the target sounds and the noise were from the same gender speakers than when from different gender speakers (Cooke et al., 2008).

Some factors connected with learners’ backgrounds have been found to cause individual differences in L2 speech perception in noise. One of them is L1 interference. The study of Garcia Lecumberri et al. (2008), in which eight groups of non-native listeners finished English intervocalic consonants perception test in quiet and six noise types, reported that strong L1 interference was observed both from the sound system and from the L1 orthography. There was a general tendency for listener groups from languages closer to English to perform better than those from more distant languages. However, it has been revealed that even learners with the same L1 have also been shown to perform differently in their L2 speech perception if they have different amounts of L1 use. MacKay et al. (2001), in an attempt to examine the roles of L1 use and age of arrival on native Italian participants’ identification of English consonants in pink noise, revealed that a native-like identification of L2 consonants can be achieved only if L2 learning begins early in life and the L1 is used relatively seldom.

Another important factor that draws researchers’ interest is the amount of experience with the target L2. Speech perception may become more native-like as a function of L2 experience. Differences in the degree, type, quality, and time of exposure to a language often give rise to differences in familiarity with linguistic patterning at all levels, from acoustic to pragmatic (Garcia Lecumberri and Cooke, 2006). Limited linguistic experience could result in a deterioration of non-native speech perception under adverse listening conditions, and increasing experience often had a tight link with a reduced masking effect of noise (Mayo et al., 1997). For Chinese English learners, Mi et al. (2013) reported that even a relatively short experience (1–2 years) in a native English environment could significantly improve Chinese listeners’ vowel identification in English multi-talker babble, although such improvement was not observed in quiet and long-term speech-shaped noise conditions.

To sum up, with regard to the effects of noise on L2 speech processing, previous research has focused mainly on the differences between L1 and L2 listeners in different types of noise at different SNRs, and has identified some factors that can cause variance among L2 listeners, such as L1 background, L2 experience, and amount of L1 use. It should be pointed out that while L2 listeners’ performance in noise at phoneme, word, and sentence levels has been extensively examined in earlier studies, their performance at discourse level remains unknown, which is worthy of deeper exploration considering its practical value in everyday communication.

Another feature of previous research is the insufficient attention paid to the roles of cognitive factors that may lead to individual differences in L2 listening in adverse conditions. Cooke et al. (2008), in their summary of potential masking effects for listeners, maintained that IM has multiple facets, i.e., misallocation of audible masker components, competing attention of masker, higher cognitive load and interference from “known language” masker. Therefore, it is plausible to predict that L2 listeners’ performance in noisy conditions should be tightly connected with their cognitive capacity. In particular, the capacities for sustained attention (Thompson et al., 2017) and working memory (Ingvalson et al., 2015) have been found to play important roles in L1 speech understanding in noise. However, too little is known at present to ascertain the effects of cognitive factors in L2 listening in noise.

In light of the above considerations, the current study approached English listening comprehension in noisy conditions among Chinese college students by looking into the role of Kolb’s learning styles, which are assumed to have links with specific preferences for certain cognitive processes, in listening comprehension of English conversations in different types of noise.

### Kolb’s Learning Styles and L2 Learning

Kolb’s theory of learning styles is based on his experiential learning theory (ELT), and has been widely employed by both researchers and practitioners (Dörnyei, 2005, p. 129; Metallidou and Platisidou, 2008). In ELT, learning is “the process whereby knowledge is created through the transformation of experience. Knowledge results from the combination of grasping and transforming experience” (Kolb, 1984, p. 41). Grasping experience pertains to the process of taking in information, and transforming experience is how individuals interpret and act on that information (Passarelli and Kolb, 2012). For grasping an experience, learners usually employ one of the two dialectically related modes: concrete experience (CE) or abstract conceptualization (AC), while for transforming an experience, they rely on one of the two dialectically related modes: reflective observation (RO) or active experimentation (AE). These four modes occur in a recursive process, resulting in an ideal four-stage learning cycle. Specifically, CEs serve as the basis for observations and reflections, through which the experiences are subsequently assimilated and distilled into abstract concepts. Then new hypotheses for action are drawn and actively tested, thereby assisting the creation of new experiences.

Learners spiral through the learning cycle in accordance with their unique preferences for these dialectic learning modes. Every learner has a general tendency to learn either through CE or through AC when grasping an experience, as well as a tendency to learn either through AE or through RO when transforming an experience. These preferences for certain learning modes are classified as four learning styles, i.e., *divergent* (CE/RO), *assimilative* (AC/RO), *convergent* (AC/AE), and *accommodative* (CE/AE). Divergers are able to view specific situations from different perspectives and combine many relationships into a meaningful whole; assimilators are good at inductive reasoning, creating theoretical models, and assimilating disparate observations into an integrated explanation; convergers are skillful at decision making, problem solving, and the practical application of ideas; accommodators prefer doing things, carrying out plans and tasks, or getting involved in new experiences (Kolb, 1984).

The Kolb Learning Style Inventory (LSI), designed in accordance with the ELT framework, is the most commonly used instrument for assessing learning styles in research and teaching (Newton and Miah, 2017) and has been increasingly applied to L2 research. Some studies based on the LSI reported that students majoring in a foreign language preferred diverging learning style (e.g., Kolb, 1981; Noguera and Wageman, 2011). The LSI might also be a good predictor of L2 academic performance (e.g., Castro and Peck, 2005; Chermahini et al., 2013). Furthermore, different learning styles have been found to have different links with certain specific learning tasks. For example, divergent learning style preference could predict learners’ performance on phonological tasks and semantic tasks, while accommodative learning style preference predicted their performance on syntactical tasks (Andreou et al., 2008; Wang and Dai, 2013). Among the Chinese college students examined in Yang et al. (2016), assimilative and divergent learning styles were more likely to facilitate developing native-like patterns of using acoustic cues for English vowel perception than convergent and accommodative learning styles.

More important, some differential performance observed in learners with different learning styles can be attributed to certain cognitive processes (An and Carr, 2017). For example, while the concrete versus abstract dichotomy on the dimension of experience grasping could be explained using expert-novice differences, learners’ preference on the dimension of experience transformation should be interpreted in terms of self-regulation of attention and inhibition. Learners who are more reflective and less impulsive are more attentive, and more likely to self-regulate and inhibit distractions. Consequently, they are good at “effortful control” by regulating attention and suppress impulses in learning activities.

In summary, as pointed out by Kolb and Kolb (2005), the matching between learning contexts and learning styles results in enhanced L2 performance. However, it remains unknown whether learning styles based on preferences for cognitive processes can contribute to L2 listening comprehension in adverse listening conditions, which was to be examined in the present study.

### The Current Study

As postulated in ELT, the process of learning from experience is an essential part of human activity everywhere all the time (Passarelli and Kolb, 2011), implying that obtaining information through listening activities in noise is a typical example of experiential learning. Accordingly, the process of listening comprehension in noise could be explained in terms of Kolb’s learning cycle. Learners first grasp speech signals through CEs of listening activities in noise (CE), and further analyze and interpret the information carried in these signals via reflection (RO). Then the results of reflection are distilled to build abstract representations of the information (AC), which are actively tested and applied to new experiences (AE) of listening activities in noise.

Therefore, it is plausible to assume that when handling listening tasks in a noisy condition, learners with different learning styles should have special preferences for certain learning modes in their learning cycle, which may ultimately lead to differential listening results. In the present study, we hypothesized that the effects of different types of noise on English listening comprehension of Chinese college students should be closely related to their learning styles. To examine this hypothesis, the LSI and a listening test on English conversation comprehension in different listening conditions were administered to Chinese college students who learned English as a foreign language (EFL).

We chose to explore listening performance in noise among Chinese college students based on the following consideration. Although English learning in China is generally characterized as formal learning in the classroom, English communication in various environments has become increasingly necessary with the development of China. However, Chinese EFL students are always confronted with enormous difficulties in adverse conditions given that they cannot handle English listening by utilizing their experience with Mandarin Chinese, since the two languages have substantial differences in many aspects.

Generally, Mandarin Chinese is a typical ideographic language based on words, while English is an alphabetic language based on sounds (Richards and Schmidt, 2002). Phonologically, Chinese is differentiated from English not only by vowels and consonants (e.g., Jia et al., 2006; Duanmu, 2007; Rattanasone and Demuth, 2014), but also by stress and intonation patterns (Duanmu, 2007; Guo and Shi, 2011; Luo et al., 2015). Rhythmically, Mandarin Chinese is a syllable-timed language, whereas English is regarded as a typical stress-timed language (Lin and Wang, 2005), and this difference has been described as that between “machine-gun” rhythm and “Morse code” rhythm. Moreover, unlike English, Chinese has lexical tones which can be used to distinguish word meanings (Duanmu, 2007; Shi et al., 2017). In addition, there are noticeable differences in grammar between the two languages, such as tense (Lin, 2015) and word order (e.g., Yuan and Dugarova, 2012).

As can be seen, when listening to English in adverse conditions, Chinese students should have new experiences different not only from those for listening tasks in the quiet classroom, but also from their Chinese listening experiences in everyday life. Exploring these experiences among Chinese students in association with their learning styles is not only important to understand how different elements in noisy environments are able to cause detrimental effects on their listening performance, but also meaningful to reveal the contribution of learning styles to listening performance in various conditions.

Therefore, the current study was designed to answer the following two questions:

(1) Does Chinese students’ English listening performance in different types of noise vary according to their learning styles?

Four types of listening conditions were set up for the English listening comprehension test, i.e., quiet, white noise, Chinese two-talker babble, and English two-talker babble. White noise is quasi-stationary and is often assumed to cause EM (e.g., Arbogast et al., 2005), while two-talker babble can produce extensive IM as well as EM (Cainer et al., 2008; Calandruccio et al., 2010). Since different amounts of EM and IM effects were involved in the four listening conditions, the cognitive tasks the students had to handle were different in difficulty. Given that certain learning styles do correlate more highly than others with desired aspects of language tasks in specific settings, we expected that the listening performance of the students in the four listening conditions should be affected differently by their learning styles.

More specifically, since listening in quiet was able to manifest the competence for speech understanding in an ideal condition that did not pose difficult challenges to all listeners, students with different styles should have similar performance. As EM can cause a loss of signal components (Garcia Lecumberri et al., 2010), which should be equal for all listeners, students with different learning styles would reach the same comprehension level in white noise. However, in English babble and Chinese babble involving IM, students with different learning styles should have differential performance because they might deal with the interference from the masking in different ways.

(2) If there is a link between Chinese students’ English listening comprehension in noise and their learning styles, then which of the four learning modes based on ELT should be essential to their performance? According to ELT, every learner employs each learning mode to a certain degree when spiraling through the learning cycle. If a certain type of learning style has been found to correlate tightly with English listening comprehension in a specific noisy condition, then one or two learning mode(s) associated with the style should contribute to this correlation. We predicted that the students’ preferences for the learning modes on the dimension of experience transformation would result in different comprehension levels, because they are closely related to the preferences for cognitive processes such as attention and inhibitory control, which are critical for speech understanding in noise.

## Materials and Methods

### Participants

A total of 89 sophomore students participated in the present study, including 42 male students and 47 female students with ages ranging from 19 to 22 (*M* = 19.60, *SD* = 1.01). They were majors of science or engineering and learned English as a foreign language. Based on their responses to Kolb’s (1985) Learning Style Inventory, 26 of the participants were classified as divergers, 24 as assimilators, 22 as convergers, and 17 as accommodators. They had similar basic competence in English listening comprehension, since there were no differences in their listening comprehension in quiet as shown in the next section. All the participants reported no history of speech or hearing disability.

### Materials

#### Kolb’s 1985 Learning Style Inventory

The Kolb’s (1985) Learning Style Inventory is one of the most widely used instruments measuring learning styles in higher education (Healey and Jenkins, 2000). The inventory in the present study was based on a Chinese version provided by Hay Group in order to minimize the impact of variation in English proficiency among the participants. It included 12 short statements about different learning situations, and the participants responded by ranking four sentence endings corresponding to the four learning modes, i.e., CE, RO, AC, and AE (see **Appendix** for example items). The score for each mode was obtained by adding up the forced ratings of the 12 statements, based on which the combination scores of AC-CE and AE-RO were calculated. A higher AC-CE score showed a comparatively greater inclination for abstractness (AC) and lesser inclination for concreteness (CE), while a higher AE-RO score suggests a preference for action (AE) over reflection (RO). The participants’ learning style types were then determined by matching these two scores with the learning style type grid provided by the inventory.

An exploratory factor analysis was performed to verify its construct validity. As in earlier studies (e.g., Yahya, 1998; Metallidou and Platisidou, 2008; Yang et al., 2016), it was based on a principal component analysis with varimax rotation and yielded the results as shown in **Table 1**.

**Table 1:** Results of exploratory factor analysis on Kolb’s (1985) Learning Style Inventory.

Scale	Factor 1	Factor 2
AE	-0.94	
RO	0.76	
AC		0.88
CE		-0.83
Eigenvalue	1.71	1.31
Variance%	42.64	32.62
Cumulative%	75.26


Furthermore, the Cronbach’s alpha coefficients of the scales of the four learning modes (0.80, 0.74, 0.75, 0.81, respectively) were generally in line with those from Smith and Kolb (1986, p. 97) (0.82, 0.73, 0.83, 0.78, respectively) and Li and Armstrong (2015) (0.75, 0.79, 0.81, 0.75, respectively). The correlation matrix in **Table 2** shows that the AC scale was negatively correlated with the CE scale, as was the AE scale with RO scale. The two combinations, i.e., AC-CE and AE-RO, were essentially independent of each other. There were only low or no correlations between AE-RO and CE, AC, or between AC-CE and AE, RO, respectively. All these results were consistent with the framework of ELT.

**Table 2:** Scale intercorrelations of Kolb’s (1985) Learning Style Inventory.

Scale	CE	RO	AC	AE	AE-RO
CE	—	-0.06	-0.48^∗∗^	-0.38^∗∗^	-0.26^∗^
RO		—	-0.22^∗^	-0.49^∗∗^	-0.83^∗∗^
AC			—	-0.20^∗^	-0.02
AE				—	0.90^∗∗^
AC-CE	-0.87^∗∗^	-0.09	0.85^∗∗^	0.15	0.14


*^∗^*p* < 0.05, ^∗∗^*p* < 0.01 (two tailed).*

#### The EFL Listening Comprehension Test

Materials for the English listening comprehension test included four pairs of conversations in American English, and each pair of them was to be randomly presented to the participants in one of four listening conditions. The content of the conversations covered some common topics in daily life, each lasting about 100 s. In each listening condition, there were one conversation with four multiple-choice questions, and one with three questions to test participants’ comprehension. According to the results of a pilot study with 100 students who had the same backgrounds as the participants in the current experiment, the Cronbach’s alpha for each of the four pairs of conversations was 0.59, 0.58, 0.59, and 0.62, and the total Cronbach’s alpha for all conversations reached 0.83. Moreover, the results of repeated measure ANOVA reported that no significant differences in the scores of the listening comprehension were found among the four pairs of conversations.

The four listening conditions included quiet, white noise, Chinese two-talker babble, English two-talker babble. The babble was produced by adding together amplitude-equalized utterances from two speakers. In the Chinese two-talker babble background, a male was reading aloud a report about Arctic climate change, while a female was reading a passage introducing the geography of east Africa, both speaking Mandarin Chinese. In the English two-talker babble background, a male and a female, who were both native speakers of American English from the United States, were reading in English the same materials as in the Chinese two-talker babble. Furthermore, the English conversations for listening comprehension in the three noisy conditions were presented at a fixed SNR of +3 dB determined according to the results of pilot studies, with the noise starting one second prior to the onset of the conversation and ceasing at the end of the conversation.

### Procedure

The participants first responded to the LSI and then completed the EFL listening comprehension test in different conditions. For the sake of better understanding of the tasks, the instructions for the listening comprehension test were given in Chinese. The test was carried out on computers using E-Prime 2.0, and the listening materials were presented over Sennheiser HD280 PRO headphones at a comfortable level. There was first a practice block in which four sample conversations for the four listening conditions were presented respectively. Then the actual test began. The four pairs of conversations were randomly presented in four blocks, one for a listening condition. The participants’ performance in quiet was always measured first, and then followed by the three noisy conditions presented in random order among the participants.

In each listening condition, the participants first listened to a conversation, and then responded to multiple-choice questions designed for the conversation. They were asked to input the number of their choice for each question on the keyboard. The next conversation would not appear until the participants had finished the current conversation and pressed the SPACE bar to continue. When the two conversations in a condition were completed, there was a short break of 10 s. The procedure was self-paced with no limit on time to respond, but no pauses were permitted during a block. The tasks for each condition needed 4–5 min to finish, and all conditions were tested in a single session. The participants’ listening performance in each condition was assessed according to the percentage of correct responses to the multiple-choice questions.

### Data Analysis

Analyses were conducted within the statistical software R (R Development Core Team, 2017). Linear mixed-effects models based on the *lmer* function of the *lme4* package (Bates et al., 2017) were performed to assess the effects of noise types and learning styles on English listening comprehension. In these models, listening score was used as the dependent variable, type of learning style, listening condition, and their interaction as fixed factors with listeners as a random factor. To examine the main effect of fixed factors, a model including the fixed factor of interest was compared with the same model without such a factor. For *post hoc* analysis on the mixed effect models, pairwise comparisons were performed using the *lsm* function of the *lsmeans* package (Russell, 2017).

Furthermore, multiple regressions were also conducted using the *lm* function from the *stats* package to identify the learning mode(s) that could best predict English listening comprehension performance in different listening conditions. In addition, standardized coefficients of the multiple regressions were obtained using the function of *lm.beta* from the *QuantPsyc* package (Fletcher, 2012). An alpha significant level of 0.05 was set for all statistical tests.

## Results

### Learning Styles and English Listening Comprehension in Different Listening Conditions

With regard to the participants’ English listening comprehension scores in different listening conditions, the mixed-effects model analysis indicated that there was a significant main effect for listening condition [χ^2^(3) = 58.69, *p* < 0.001]. Pairwise comparisons showed that the students’ listening scores in quiet and in white noise were similar to each other (*M* = 76.73, *SD* = 19.19; *M* = 70.95, *SD* = 22.01), and were both significantly better than those in the two babble conditions (*M* = 60.67, *SD* = 26.92; *M* = 56.98, *SD* = 24.41) (*p* < 0.01 for all comparisons), while there were no differences in the latter two conditions.

**FIGURE 1:**
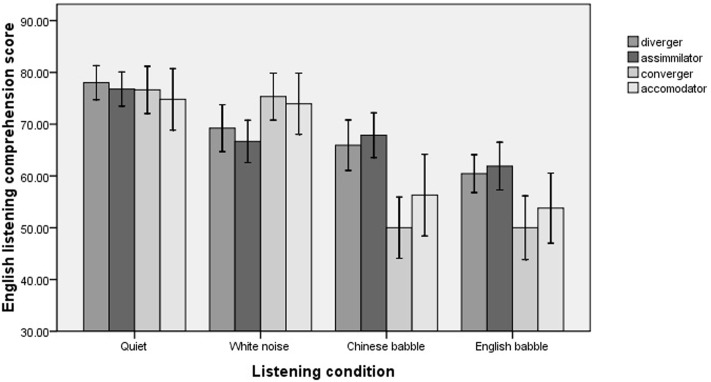
Participants’ English listening comprehension in different listening conditions.

**Figure 1** illustrates the listening comprehension performance of the participants in different listening conditions when their learning styles were taken into consideration. The mixed-effects model analysis revealed that while no main effect for learning style was observed [χ^2^(3) = 1.76, *p* = 0.62], there was a significant interaction between learning style and listening condition [χ^2^(9) = 18.86, *p* = 0.026].

To further explore the interaction between listening condition and learning style, paired comparisons were conducted. The results indicated that in Chinese babble, the assimilators performed better than the convergers (*p* = 0.047), and the divergers were better to a certain extent than the convergers (marginally significant, *p* = 0.085). In the other three conditions, there were no differences among the four groups.

Furthermore, it was found that different learning styles led to different performance patterns across the four listening conditions. Specifically, for both the divergers and the assimilators, their performance remained stable and the only differences were observed between their scores in quiet and in English babble (*p* < 0.05 for both comparisons). By contrast, for both the convergers and the accommodators, their scores in Chinese babble and English babble were much lower than those in quiet and in white noise (*p* < 0.05 for all comparisons), while there were no significant differences between their scores in the latter two conditions.

In summary, noise types had different effects on the participants with different learning styles. The assimilators and the divergers were relatively better in Chinese babble, and they also had stable performance across the three noisy conditions, while the convergers and the accommodators suffered significantly from the IM produced by both Chinese babble and English babble.

### Learning Modes and English Listening Comprehension in Different Listening Conditions

According to ELT, a learner’s learning style can be derived from the combination of his/her preferred learning modes on the two bipolar dimensions, i.e., AC-CE for grasping an experience and AE-RO for transforming an experience. Then which dimension is more critical to English listening comprehension in noise? As shown in the results obtained above, the assimilators (AC/RO) and the divergers (CE/RO) both had similar performance across the three noisy conditions, while the convergers (AC/AE) and the accommodators (CE/AE) had significant loss in Chinese babble and English babble conditions. The assimilators and the divergers, though different in their preferences on the AC-CE dimension, both relied on RO on the AE-RO dimension. By contrast, the convergers and the accommodators, though opposite to each other on the AC-CE dimension, both depended on AE on the AE-RO dimension. It was predicted, therefore, that the participants’ preferences on the AE-RO dimension, i.e., the ways of transforming an experience, should be essential to their listening performance.

To test this prediction, the correlations between the participants’ performance in the four listening conditions and their learning modes preferences were first examined. As can be seen from **Table 3**, both their scores in Chinese babble and English babble were significantly related to the AE-RO dimension, implying that the more they preferred the RO mode, the better their performance in the two babble conditions. Moreover, their scores in the three noisy conditions all closely correlated with their performance in quiet. If the participants’ scores in quiet could manifest their basic competence in English comprehension, then this competence would significantly affect their performance in the noisy conditions.

**Table 3:** Correlations between participants’ performance in the four listening conditions and their learning mode preferences.

	1	2	3	4	5	6
(1) Quiet	—	0.35^∗∗^	0.23^∗^	0.50^∗∗^	0.05	-0.10
(2) White noise		—	0.41^∗∗^	0.48^∗∗^	0.06	0.11
(3) Chinese babble			—	0.32^∗∗^	0.07	-0.24^∗^
(4) English babble				—	-0.04	-0.26^∗∗^
(5) AC-CE					—	0.14
(6) AE-RO						—


*^∗^*p* < 0.05, ^∗∗^*p* < 0.01.*

Given the complex interrelations shown above, multiple regression analyses were performed to identify the mode(s) that could best predict the participants’ performance in each listening condition. The results are presented from **Tables 4**–**7**.

**Table 4:** Beta-weights of AE-RO, AC-CE as the predictors of English listening comprehension in quiet.

Predictor	Stand. Beta	*t*	*p*
AE-RO	-0.10	-0.96	0.339
AC-CE	0.06	0.56	0.576


**R ^2^* = 0.01, *F* = 0.55, *p* = 0.58.*

**Table 5:** Beta-weights of AE-RO, AC-CE and comprehension scores in quiet as the predictors of English listening comprehension in white noise.

Predictor	Stand. Beta	*t*	*p*
AE-RO	0.14	1.36	0.177
AC-CE	0.02	0.20	0.839
Scores in quiet	0.36	3.61	0.001


**R^2^* = 0.14, *F* = 4.78, *p* = 0.004.*

**Table 6:** Beta-weights of AE-RO, AC-CE and comprehension scores in quiet as the predictors of English listening comprehension in Chinese babble.

Predictor	Stand. Beta	*t*	*p*
AE-RO	-0.23	-2.25	0.027
AC-CE	0.09	0.91	0.367
Scores in quiet	0.21	2.00	0.048


**R* square = 0.11, *F* = 3.53, *p* = 0.018.*

**Table 7:** Beta-weights of AE-RO, AC-CE and comprehension scores in quiet as the predictors of English listening comprehension in English babble.

Predictor	Stand. Beta	*t*	*p*
AE-RO	-0.21	-2.27	0.026
AC-CE	-0.03	-0.34	0.738
Scores in quiet	0.48	5.21	<0.001


**R^2^* = 0.29, *F* = 11.77, *p* < 0.001.*

As can be seen from **Table 4**, both the AE-RO and the AC-CE dimensions did not have noticeable effects on the participants’ performance in quiet. It should be noted that for listening comprehension in the three noisy conditions, the participants’ performance in quiet was also included in the regression analyses as one of the predictors due to its strong correlations with their listening performance in the noisy conditions. The results indicated that in white noise, only their scores in quiet could play a substantial role in their listening comprehension (see **Table 5**). However, in Chinese babble and English babble, both AE-RO and the scores in quiet reliably predicted their listening performance, suggesting that the higher their competence in quiet and the more they preferred the RO mode, the better they performed in these two conditions (see **Tables 6**, **7**).

## Discussion

The current study was an attempt to explore whether the effects of different types of noise on English listening comprehension among Chinese college students would vary with their learning styles based on Kolb’s (1985) Learning Style Inventory. To answer the two research questions, the findings were analyzed and discussed as follows.

In general, the students’ listening comprehension scores in the two babble conditions were both significantly lower than their scores in quiet, suggesting that their listening ability was markedly impaired by IM. However, the difference between their scores in white noise and those in quiet did not reach a significant level. Moreover, their performance in white noise was also better than in Chinese two-talker babble and English two-talker babble at the present SNR of +3 dB. These findings suggested that the impairment caused by EM was less than IM, completely consistent with the findings from previous research at phoneme, word, and sentence levels (e.g., Shimizu et al., 2002; Garcia Lecumberri and Cooke, 2006; Cutler et al., 2008; Kilman et al., 2014). It should be noted that the students’ performance in Chinese babble was similar to that in English babble, as they were familiar with both languages (Garcia Lecumberri and Cooke, 2006). In addition, the students’ performance in the three noisy conditions was tightly related to that in quiet, implying that the basic competence in listening comprehension exhibited in quiet did play a substantial role in listening comprehension in various noisy conditions.

More important, when learning styles were taken into account, the students’ performance patterns in different listening conditions manifested non-negligible individual differences. First of all, the students all had similar performance in quiet, suggesting that their English listening comprehension competence was exactly at the same level. However, the divergers and the assimilators were both better than the convergers in Chinese babble. At the same time, the divergers and the assimilators performed similarly across the three noisy conditions, exhibiting a relatively stable listening capacity, while both the convergers and the accommodators suffered a larger performance deficit in English two-talker babble and Chinese two-talker babble compared with their performance in quiet and in white noise.

Therefore, at the current SNR of +3 dB, different types of noise were able to cause differential performance in English listening comprehension among students with different learning styles. The possible explanation lies in the different masking effects involved in the three noisy conditions. White noise is known to produce pure EM (Arbogast et al., 2005), which may cause a loss of signal components, so listeners have to employ partial information to interpret the speech signals (Cooke et al., 2008). Consequently, the students in the current study, in spite of their differences in learning style preference, were confronted with the same difficulties at the periphery of the auditory system. Considering that they had similar listening comprehension competence as shown in quiet, there should be no significant differences in utilizing partial information to interpret the signals when they were listening in white noise.

By contrast, two-talker babble is known to induce extensive IM, portions of which could be mistakenly perceived as being part of the target sounds to be identified (Van Dommelen and Hazan, 2010). According to Cooke et al. (2008), information masking effects may arise because of misallocation of audible masker components to target (or misallocation of target elements to masker), competing attention of masker, higher cognitive load and interference from “known language” masker. In the present study, English listening comprehension in both English two-talker babble and Chinese two-talker babble was relatively challenging to Chinese EFL learners, and how the students handled the interference from the masker sounds would determine the extent their performance was impaired compared to their competence exhibited in quiet. So, the divergers and the assimilators, who could more effectively distinguish the target speech from the masker sounds, suffered less from the masking effect than the convergers and the accommodators in comparison with their performance in quiet. Then why were the Chinese and English babble conditions able to cause problems to the convergers and the accommodators, but not to the divergers and the assimilators?

According to the findings of the current study, the performance of the students was closely related to their preferences between the two learning modes on the experience-transforming dimension, i.e., AE and RO. The more they favored the RO mode, the better they performed in listening. By contrast, the more they depended on the AE mode, the worse their listening performance. Meanwhile, their preferences between the other two modes on the experience-grasping dimension, i.e., AC and CE, brought about little effects on their listening. These findings indicated that when listening to English comprehension in two-talker babble conditions, how one transforms the rich information carried in speech signals from different sources is more crucial than how he/she grasps speech signals from their listening experience.

The inherent cognitive features exhibited in these two learning modes might account for the individual differences in English listening comprehension in babble noise. According to An and Carr (2017), individual preferences on the experience-transforming dimension can lead to differential performance in cognitive processes, such as attentional and inhibitory control. Reflective learners are more attentive and more able to self-regulate and inhibit distracting interference than impulsive learners. These advantages of the RO learning mode in cognitive processes have been echoed in views from research on learning behaviors. For example, reflective learners are more likely to thoroughly collect data and reflect on them before reaching definitive conclusions (Honey and Mumford, 1982). Moreover, the process of reflection enables learners to gain advantages in metacognitive activities (Palladino et al., 1997), such as considering the plans prior to engaging in a task, the assessments and adjustments while they work, and the revisions afterward (Driscoll, 1994; Ertmer and Newby, 1996), which subsequently result in better performance in learning success (Maddox and Chandrasekaran, 2014).

More important, the cognitive mechanisms linked to attention and inhibition have been found to play critical roles in speech understanding in noise. For example, Thompson et al. (2017) reported that their participants’ improvement in speech-in-noise performance was related to their gains in attention. Ingvalson et al. (2015) also revealed that improvement in working memory can effectively facilitate speech perception in noise.

As far as the present study is concerned, it could be assumed that, when it comes to English listening comprehension in babble noise, the strengths of reflective learning should allow the students with assimilative and divergent styles to outperform the students with other styles. The assimilators and the divergers tended to carefully analyze the target and masker sounds, and actively compare, evaluate and make adjustment to their information processing on the basis of their cognitive and metacognitive advantages. Thus they were able to effectively track the target sounds by focusing on the information carried in the target sounds and ignoring the interfering information from the masker speech, thereby achieving higher accuracy in their comprehension.

Unlike RO, the mode of AE might bring more problems to the students with convergent and accommodative styles in their English listening. According to Furnham (1992), convergers often make decisions too quickly and solve the wrong problem, while accommodators tend to involve themselves in trivial activities. Consequently, when listening to English conversation in babble noise, these two types of students were particularly prone to be drawn away by babble noise because of lacking the motives to closely analyze and evaluate what they had heard, which caused cognitive resources to be allocated to both the target and the masker. Given that their attention was based on limited resources, a higher cognitive load inevitably gave rise to difficulties in tracking the target (Cooke et al., 2008), resulting in poorer listening comprehension performance.

The findings of the current study may shed light on foreign language teaching and learning in coping with listening comprehension in adverse conditions. According to Ehrman (1996), the effective matching of task demands and learning styles will lead to an adaptive competence. As learning style is a dynamic state on the basis of synergistic transactions between the person and the environment instead of a crystallized psychological trait (Passarelli and Kolb, 2011), teachers may consider helping students apply learning strategies or skills based on the RO mode to their listening practice in noisy conditions. For instance, deliberately viewing things from different perspectives and practicing information skills with regard to sense-making, information gathering and information analysis can facilitate the development of reflective learning (Kolb and Kolb, 2008).

The present study leaves unanswered a number of questions that could be addressed in future research. First, the study just examined the role of learning styles in listening comprehension in different types of noise at a fixed SNR. Whether the role will change with SNRs remains unknown, as previous studies have shown that listeners performed differently in noise at different SNRs (Garcia Lecumberri et al., 2010). Second, IM could be manipulated in a number of ways, such as gender of the competing talker, spatial separation of the competing speech, linguistic content of the competing speech, etc. (Calandruccio et al., 2010), which would lead to various interesting and informative patterns of masking effects. Further looking into these dimensions would greatly add to the value of the findings from the current study.

Finally, it should be acknowledged that the assumed cognitive advantages of the RO learning mode in FL listening in noise need to be further examined. Although the basic cognitive abilities for attentional and inhibitory control have been found to be tightly linked to speech understanding in noise, it remains unclear to what extent the preferences for such cognitive control, i.e., differences in learning styles, can invoke corresponding inhibitory and attentional processes in L2 speech perception in noise. This issue might be explored in a neurological approach. For example, researchers recently began to apply functional magnetic resonance imaging (fMRI) and electroencephalography (EEG) techniques to L1 or L2 speech perception in noise (e.g., Wong et al., 2009; Dole et al., 2014; Thompson et al., 2016). By revealing differences in the neurological activities underlying listening comprehension performance in noisy conditions among L2 learners with different learning styles, these techniques are able to offer more interesting insights into the contributions of cognitive factors to the parts played by learning styles. Notwithstanding these limitations, the results of the current study may provide a useful starting point for understanding the important role of learning styles in L2 listening comprehension in noise.

## Conclusion

Our findings showed that the effects of different types of noise on English listening comprehension among Chinese college students varied depending on their learning styles. Specifically, assimilative and divergent learning style preferences were more beneficial for English listening comprehension in noise than convergent and accommodative learning styles. In addition, how students transformed the information carried in speech signals presented in babble noise was more crucial than how they grasped the signals in their listening experience. More important, for transforming listening experience in babble noise, the mode of RO had a facilitative effect on listening performance, whereas the mode of AE was more connected with listening difficulties.

## Ethics Statement

This study was carried out in accordance with the recommendations of the Ethic Committee of School of Foreign Languages, Shanghai Jiao Tong University with written informed consent from all subjects. All subjects gave written informed consent in accordance with the Declaration of Helsinki. The protocol was approved by the Ethic Committee of School of Foreign Languages, Shanghai Jiao Tong University.

## Author Contributions

XY designed the study; XY and YZ prepared the materials; MJ conducted the experiment; XY and MJ analyzed the data; XY, MJ, and YZ wrote the manuscript.

## Conflict of Interest Statement

The authors declare that the research was conducted in the absence of any commercial or financial relationships that could be construed as a potential conflict of interest.

## APPENDIX

**Table A1:** Examples from Kolb’s Learning Style Inventory (1985).

1. When I learn:	I like to deal with my feelings	I like to watch and listen	I like to think about ideas	I like to be doing things
7. I learn best from:	Personal relationships	Observation	Rational theories	A chance to try out and practice
